# Optical probing of ultrafast laser-induced solid-to-overdense-plasma transitions

**DOI:** 10.1038/s41377-024-01444-y

**Published:** 2024-05-08

**Authors:** Yasmina Azamoum, Georg Alexander Becker, Sebastian Keppler, Guillaume Duchateau, Stefan Skupin, Mickael Grech, Fabrice Catoire, Sebastian Hell, Issa Tamer, Marco Hornung, Marco Hellwing, Alexander Kessler, Franck Schorcht, Malte Christoph Kaluza

**Affiliations:** 1 GSI Helmholtzzentrum für Schwerionenforschung, Planckstr. 1, 64291 Darmstadt, Germany; 2 Helmholtz Institute Jena, Fröbelstieg 3, 07743 Jena, Germany; 3 Institute of Optics and Quantum Electronics, Friedrich-Schiller-Universität Jena, Max-Wien-Platz 1, 07743 Jena, Germany; 4 CEA-CESTA, 15 Avenue des Sablières, CS60001, 33116 Le Barp, Cedex France; 5 Institut Lumière Matière, UMR 5306 - CNRS, Université de Lyon 1, 69622 Villeurbanne, France; 6 LULI, CNRS, CEA, Sorbonne Université, Institut Polytechnique de Paris, Palaiseau, France; 7 Université de Bordeaux-CNRS-CEA, CELIA, UMR 5107 Talence, France

**Keywords:** Laser-produced plasmas, Ultrafast photonics

## Abstract

Understanding the solid target dynamics resulting from the interaction with an ultrashort laser pulse is a challenging fundamental multi-physics problem involving atomic and solid-state physics, plasma physics, and laser physics. Knowledge of the initial interplay of the underlying processes is essential to many applications ranging from low-power laser regimes like laser-induced ablation to high-power laser regimes like laser-driven ion acceleration. Accessing the properties of the so-called pre-plasma formed as the laser pulse’s rising edge ionizes the target is complicated from the theoretical and experimental point of view, and many aspects of this laser-induced transition from solid to overdense plasma over picosecond timescales are still open questions. On the one hand, laser-driven ion acceleration requires precise control of the pre-plasma because the efficiency of the acceleration process crucially depends on the target properties at the arrival of the relativistic intensity peak of the pulse. On the other hand, efficient laser ablation requires, for example, preventing the so-called “plasma shielding”. By capturing the dynamics of the initial stage of the interaction, we report on a detailed visualization of the pre-plasma formation and evolution. Nanometer-thin diamond-like carbon foils are shown to transition from solid to plasma during the laser rising edge with intensities < 10^16^ W/cm². Single-shot near-infrared probe transmission measurements evidence sub-picosecond dynamics of an expanding plasma with densities above 10^23^ cm^−3^ (about 100 times the critical plasma density). The complementarity of a solid-state interaction model and kinetic plasma description provides deep insight into the interplay of initial ionization, collisions, and expansion.

## Abstract (web summary)

A novel, single-shot probing technique visualizes the ultrafast laser-induced solid-to-plasma transition. The entire target dynamics (ionization to overdense plasma) is elucidated by combining solid-state interaction model and kinetic plasma description.

## Graphical abstract

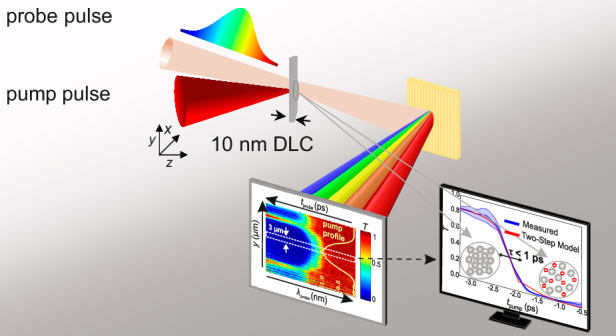

## Introduction

Ultrafast laser-solid interaction enabled major technological breakthroughs over the last half-century in low- and high-power laser regimes. In the former, controlled modifications of materials are nowadays achieved through advanced laser ablation such as micro-machining in solids^1,2^ or production of nanoparticles using laser ablation in liquids^3,4^. In the latter, ion beams with unique properties^5,6^ produced from relativistic laser-thin foil interactions paved the way to ground-breaking applications like time-resolved radiography of electric and magnetic fields in plasmas^7^, fast ignition in inertial confinement fusion^8,9^, material testing and analysis^10^, proton and carbon ion radiobiology^11,12^ and cancer therapy^13^. However, challenges need to be overcome in order to achieve better performance in laser ablation and to bring laser-based ion beams to societal applications. Certainly, the interaction between lasers and solids encompasses a multitude of fundamental processes, the interplay of which largely remains undetermined. Hence, it is crucial to clarify the interplay of these processes in the early stages of the interaction. On the one hand, this is necessary to prevent phenomena such as “plasma shielding,” which diminishes ablation efficiency^3^, and on the other hand, it is essential for the precise control of the relativistic laser-thin foil interaction. Rapid progress in laser-driven ion acceleration over the past two decades has demonstrated the production of proton energies up to ~ 100 megaelectronvolts (MeV)^14^, yet protons of ~ 200 MeV are required for radiation oncology^15^. However, theoretical predictions have anticipated the acceleration of protons with energies reaching several hundred MeV^16,17^, providing additional impetus for ongoing experimental efforts^14,18,19^.

Due to the complex nature of the interaction, several ion acceleration mechanisms can be triggered, which intricately depend on the laser pulse parameters, the properties of the target and the plasma, and their spatiotemporal evolution during the interaction. The temporal intensity profile of the laser pulse, i.e., the laser contrast, exhibits a rising edge preceding the relativistic peak and may even include a pedestal due to amplified spontaneous emission (ASE) and ultra-short pre-pulses. Thus, in real-world experiments, the target is always ionized *before* the relativistic intensities are reached, forming the so-called pre-plasma. Efficient ion acceleration may occur depending on the pre-plasma state. For instance, in the case of Radiation Pressure Acceleration (RPA)^20^ using nanometer-thin foils, one tries to minimize pre-plasma expansion at all costs because the strong pressure of the relativistic pulse is supposed to accelerate a thin layer of ions and electrons of a *non-expanded* target. Thus, RPA requires an ultrahigh laser contrast, which poses an extreme challenge for current state-of-the-art high-power lasers. In contrast, pre-plasma conditions can be tailored to optimize Target Normal Sheath Acceleration (TNSA)^21,22^ employing µm-thick foils. The electrostatic field, which accelerates the protons at the target rear side, is induced by hot electrons heated by the main laser pulse in the pre-plasma produced on the target front side. Although extensively investigated, to date, proton energies achieved in TNSA^23^ and RPA^24^ have not yet exceeded 100 MeV.

Alternative acceleration schemes proposed in^16,17^ are based on Relativistic-Induced Transparency (RIT)^25,26^. In contrast to surface acceleration as in TNSA and RPA, the laser peak penetrates a near-critical overdense pre-plasma owing to the relativistic increase of the electrons’ mass, which leads to a change in the plasma’s refractive index (RIT effect). Hence, efficient heating of the particles may occur in the now-extended interaction volume. Although appealing, the success of RIT-based schemes in experiments is challenging. Pre-expanded nanofoils are usually employed in this regime^14,18^. In this scenario, the evolution of the pre-plasma density must be tailored to the rising edge of the main pulse.

Fine-tuning the target state before the arrival of the pulse peak requires accurate modeling to identify the tunable key parameters. In conventional modeling approaches utilizing particle-in-cell (PIC) simulations^27,28^, the interaction is described starting from a pre-plasma state, which is then irradiated by the relativistic peak of the laser pulse. The initial stage of the plasma formation from the solid state by the laser rising edge is usually ignored. However, several preponderant fundamental processes occur in this early interaction stage. Among others, these include initial ionization in the solid state, conduction electron heating by the laser, electron energy-coupling to the lattice or ions, phase transitions, and collisions. Simplifying the interplay of these processes is commonly done by making strong assumptions about the complex process of pre-plasma formation. Hydrodynamic codes^29,30^ are usually employed to infer pre-plasma properties considering the laser’s rising edge and possible pre-pulses. Although these codes provide a reasonable estimate of the spatial density profiles, the distribution functions of the species, i.e., their temperatures and the ionization state, rely on approximations. For instance, only averaged physical quantities (densities, velocities, etc.) are considered in these codes, assuming the distribution functions of particles at equilibrium. This oversimplification may result in an inaccurate prediction of the pre-plasma properties and the acceleration process, motivating further experimental and theoretical investigations.

In experiments, several techniques allow us to diagnose the pre-plasma properties. For instance, the target reflectivity of the intense pulse^31^ can be used to evidence the pre-plasma formation, while monitoring the critical density surface is achieved by measuring the Doppler shift^32,33^ of the backscattered light of the pulse’s fundamental and its second harmonic from the target. However, capturing the ultrafast evolution of the pre-plasma during the steep laser rising edge, i.e., femtosecond (fs) dynamics on the nanometer (nm) scale, is challenging. Furthermore, the shot-to-shot fluctuations inherent to high-power lasers emphasize the desirability of single-shot probing diagnostics.

Optical probing is a convenient tool for investigating such plasmas. However, light with wavelength *λ* can only propagate in a plasma with electron density ${n}_{e} \,< \,{n}_{c}$ where *n*_*c*_ is the critical density given by ${n}_{c}={\omega }^{2}{m}_{e}{\varepsilon }_{0}/{e}^{2}\approx 1.11\times {10}^{21}{{\rm{cm}}}^{-3}/{[{\rm{\lambda }}({\rm{\mu m}})]}^{2}$. Then, a non-collisional, non-magnetized, and non-relativistic plasma exhibits a real-valued refractive index $\eta =\sqrt{1-{n}_{e}/{n}_{c}}$. Thus, techniques such as interferometry^34,35^ and shadowgraphy^19,36^ usually employed to monitor pre-plasma evolution are constrained to diagnose underdense plasma, or offer limited temporal and spatial resolutions in the picosecond (ps) range and micrometer scale, respectively. The nm-scale pre-plasma expansion was recently measured with reflected visible probe light from the target^37^. In contrast, the density and the temperature evolutions of plasmas that are overdense for near-infrared (NIR) light, ${n}_{e} \,>\, {10}^{21}{{\rm{cm}}}^{-3}$, could only be diagnosed using laser-driven extreme ultraviolet (XUV) or hard X-ray sources in combination with hydrodynamic codes or X-ray absorption spectroscopy with computationally demanding ab initio calculations^38,39^ (sub-ps time resolution), or using X-ray free electron lasers^40^ (fs- and nm-resolutions). Although these techniques give access to overdense plasma properties and an accurate description at the atomic level, they imply using limited-access facilities or, at least, a rather complex setup for the probe. None of the methods mentioned above reported single-shot probing.

In this article, we will exploit the fact that longer probe wavelengths, e.g., in the NIR regime, can still be used to diagnose such plasmas. When ${n}_{e} > {n}_{c}$, the probe light is primarily reflected as *η* becomes imaginary. However, a fraction of light still penetrates the plasma over the skin depth ${l}_{s}\approx c/{\omega }_{p}$, where ${\omega }_{p}=\sqrt{{n}_{e}{e}^{2}/({m}_{e}{\varepsilon }_{0})}$ is the plasma frequency. If the plasma is sufficiently thin ($< {l}_{s}$), the NIR light tunnels through and, therefore, can be detected to investigate the target’s dynamics. Besides, as ${l}_{s}\propto 1/\sqrt{{n}_{e}}$, a large electron density range can be probed in ultrathin targets. Furthermore, when the pulse is temporally chirped, the probe light allows us to investigate the plasma dynamics in single-shot measurements^41^.

To model such optical probing of thin, overdense plasma dynamics, we propose a novel and alternative approach to describe, with a limited number of postulates, the pre-plasma formation from the initial laser-target interaction, namely, the transition from the solid state to the plasma state. The further pre-plasma evolution before the arrival of the main pulse peak can be readily described using well-established PIC codes. So far, such a transition has only been investigated at lower peak intensities ($< {10}^{13}\,{\rm{W}}/{{\rm{cm}}}^{2}$), using laser pulses ($\sim \!100{\,\rm{ps}}$) with plasma dynamics reported on tens of ps time scales^42^. In contrast, an ultrafast transition was only investigated in experiments with < 100 fs-resolution^43^ using sub-picosecond laser pulses. However, to our knowledge, the ultrafast solid-to-plasma transition during a steep laser rising edge has not yet been described in detail.

Using a pump-probe approach comprising single-shot NIR probe light transmission measurements and a two-step interaction model, we report on the experimental observation of an ultrafast transition from solid to highly overdense and expanding plasma. The latter is triggered during the laser rising edge when irradiating nm-thin diamond-like carbon (DLC) foils by femtosecond laser pulses with peak intensities exceeding ${I}\sim {10}^{16}$ ${\rm{W}}/{{\rm{cm}}}^{2}$. Besides capturing the early stage of the interaction, which is relevant for laser ablation, similar parameters can willingly be employed when using a controlled pre-pulse interacting with a relativistic peak in the context of RIT, which additionally motivates this study.

## Results

**Fig. 1:**
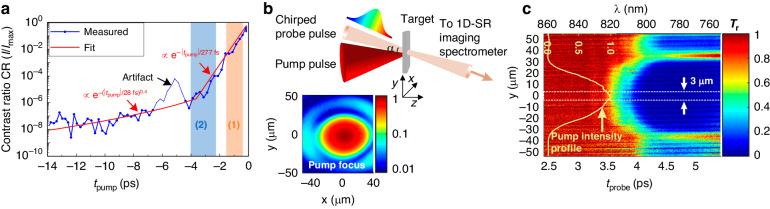
Single-shot space and time-resolved probe transmission measurement. **a** The pump laser’s temporal intensity contrast. The color-shaded regions (1) and (2) are related to the modeling discussed in Fig. 2e. The feature at ${t}_{\rm{pump}} \sim -5\,\rm{ps}$ is an artifact from the measurement, cf. Materials and methods. **b** The experimental arrangement. The pump pulse is focused at normal incidence on the target, while the probe pulse is obliquely incident under an angle $\alpha =37^{\circ}$. The inset shows the pump focus’s normalized spatial intensity profile. **c** 1D-spatially and temporally resolved relative transmission ${T}_{r}$ of the probe for a 10 nm-thick DLC foil measured using a 1D-spatially resolving (SR) imaging spectrometer and a chirped probe pulse. The wavelength on the top axis is converted into time on the bottom axis where ${t}_{{\rm probe}}=0\,{\rm ps}$ is arbitrarily chosen and corresponds to the arrival of the probe wavelength λ *≈* 920 nm. The inserted yellow curve is the normalized spatial intensity profile of the pump pulse in the transverse *y*-direction (cf. inset in **b**)

The evolution of the laser contrast of the pump pulses, used to irradiate nm-thick DLC foils, is depicted in Fig. 1a. The profile is well described by the two fitting curves in red, which will be used in the upcoming data analysis and modeling. Particularly relevant will be the steep rising edge in the time window $-3.7{\,\rm{ps}}\le {t}_{{\rm{pump}}}\le -0.2{\,\rm{ps}}$, described by the contrast ratio ${\rm{CR}}=\exp (-\left|{t}_{{\rm{pump}}}\right|/277{\,\rm{fs}})$ with an intensity profile $I={I}_{{\rm{peak}}}\times {\rm{CR}}$, where ${{I}}_{{\rm{peak}}}$ is the maximum intensity at the peak of the pulse. To ensure that the plasma is formed during this steep rising edge only (around an intensity $I\sim {10}^{12}\,{\rm{W}}/{{\rm{cm}}}^{2}$), ${{I}}_{{\rm{peak}}}$ is reduced to $\sim \!{10}^{15}\,{\rm{W}}/{{\rm{cm}}}^{2}$ so that significant ionization starts after ${t}_{{\rm{pump}}} \sim -4{\,\rm{ps}}$. As the steep rising edge covers ~5 orders of magnitude in intensity, varying the peak intensity by one order of magnitude may result in a relative time shift of the plasma formation. However, because of the particular functional form of the contrast ratio CR, apart from this shift, the temporal evolution of the plasma remains unchanged. The experimental setup is shown in Fig. 1b. The interaction region was diagnosed in the transverse *y*-direction and in time, using a temporally chirped broadband probe pulse in transmission whose different wavelengths arrive at different interaction times. With this approach, the plasma formation and evolution can be recorded within a single shot, achieving a sub-ps time resolution^44^ (see details for pulse characterization and probing in section Materials and methods).

A typical space- and time-resolved probe transmission map measured with a 1D spatially resolved imaging spectrometer through a main-pulse irradiated, 10-nm thick DLC foil as the target is shown in Fig. 1c. Such a map reveals the transition from the target being transparent to an opaque state where the probe is blocked for $\lambda$ $\lesssim \!\,820{\,\rm{nm}}$ and hence ${T}_{r}\sim 0$. The plasma profile in the transverse *y*-direction exhibits a shape and size similar to the focal spot, cf. inset of Fig. 1b, indicating that the laser’s first low-intensity Airy ring beyond the first minimum induces the wings observed in the plasma transverse profile.

In the upcoming analysis, we focus on the plasma dynamics in the central high-intensity region of the focal spot at $y=0\,{\upmu} \rm{m}$. For simplicity, in this article the particle densities are expressed as a function of the critical plasma density ${n}_{c}=1.72\times {10}^{21}{{\,\rm{cm}}}^{-3}$ for the probe wavelength $\lambda =800\,{\rm{nm}}$. We observed in our simulations that the plasma dispersion is negligible in the probe wavelength range $\lambda \approx 700-900\,{\rm{nm}}$ (cf. Supplementary materials). DLC foils of various thicknesses (5, 10, 20, 50 nm) were used as the target.

**Fig. 2:**
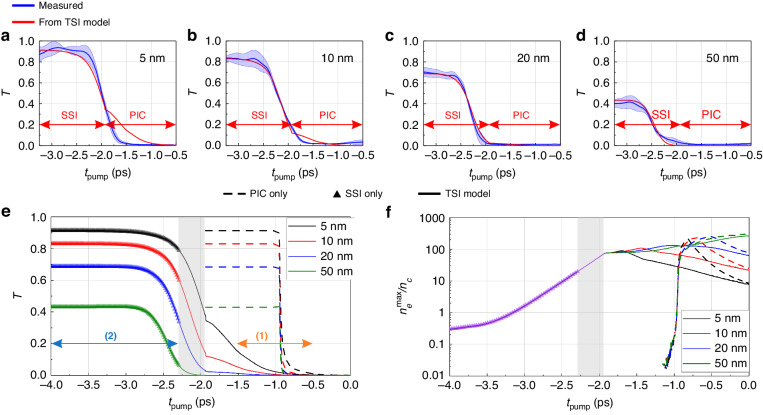
Measured and computed probe absolute transmission *T*(*t*) through the interaction region (*y* = 0 µm) induced by the pump laser pulse on DLC foils, and the calculated plasma electron densities. **a**–**d** Measured (blue) and calculated (red) $T(t)$ for DLC foils of thicknesses from 5 to 50 nm. The measurements are averaged over four shots with peak intensities ${I}_{{\rm peak}}\sim {10}^{15}-{10}^{16}\,\rm{W/{{cm}}^{2}}$. The blue shaded region is the standard deviation over all shots for each foil. The red curve is computed using the TSI model with ${I}_{\rm{peak}}\sim {10}^{15}\,\rm{W/{{cm}}^{2}}$ and plotted as a function of ${t}_{\rm{pump}}$. The measured and calculated curves are aligned at their inflection points where the relative transmission is 50 %. The red arrows delimit the time intervals for SSI and PIC steps in the TSI model. **e** Computed time-dependent probe transmission for different interaction models for different DLC foil thicknesses (5 nm: black, 10 nm: red, 20 nm: blue and 50 nm: green): the dashed line, the triangles and the solid line correspond to PIC only, SSI only (up to ${n}_{e}$ ≈ ${20n}_{c}$) and the TSI model, respectively. The blue and orange arrows correspond to the time intervals given by the shaded regions with the same colors in the laser temporal intensity profile in Fig. 1a. **f** Maximum electron densities ${n}_{e}^{\max }$ as a function of ${t}_{\rm{pump}}$ corresponding to the different simulations shown in (**e**). The density obtained from the SSI model does not depend on the foil thickness by construction and is therefore shown in violet. The shaded grey regions in (**e**) and (**f**) indicate where the extended SSI model (up to ${n}_{e}$ ≈ ${70n}_{c}$) was used

The measured absolute transmissions *T* for all DLC thicknesses are depicted as the blue lines in Fig. 2a–d (see Supplementary materials and section Materials and methods for measurements and processing details). The transmission profiles for each foil are reproducible when varying the peak intensity over one order of magnitude, which contrasts with previous works^42,43^, where the transmission dynamics varies in shape and amplitude depending on intensity. This result confirms that the plasma always forms during the steep rising edge of the pump pulse described by ${\rm{CR}}=\exp (-\left|{t}_{{\rm{pump}}}\right|/277\,{\rm{fs}})$. Varying the peak intensity only shifts the plasma formation in time, that is, by $\sim 1{\,\rm{ps}}$, but the temporal evolution remains the same. Therefore, knowledge of the absolute timing of the pump’s arrival is not required here. For better comparison, the measured transmission curves in Fig. 2a–d are shifted so that they are aligned with the calculated ones at their inflection point, that is, where the relative transmission is ${T}_{r}= 50 \, \%$. The profiles show a sub-picosecond transition from a transparent, solid-state target foil represented by the plateau ($T={T}_{0}$) at early times to an overdense plasma state at later times ($T\sim 0$).

Transmission dynamics can be characterized by the thickness-dependent transmission time $\tau \sim 400-700$ ${\rm{fs}}$ required for transmission to drop from $90\,\%$ to $10\,\%$.

As the targets are very thin (thickness $\ll \!\,\lambda$), the low measured probe transmissions ($T\sim 0.01$) imply highly overdense plasmas with electron densities ${n}_{e}\gg {n}_{c}$. To evaluate the impact of the plasma electron density on the probe transmission, we calculated the optical tunneling of the probe through a homogeneous plasma slab analytically (see Materials and methods). For a 10 nm thick plasma slab with electron density ${n}_{e}=50{n}_{c}$, this estimation leads to $T\sim 0.26$. Hence, a significant fraction of the probe intensity is expected to tunnel through the target. However, our measurements yield even lower transmission values, indicating that the plasma is highly overdense $> 50{n}_{c}$. Furthermore, assuming a plasma slab of 5 nm thickness with electron density ${n}_{e}^{{\rm{fi}}}=371{n}_{c}$, corresponding to full ionization of the DLC foil (see Materials and methods for target properties), the previous plasma slab model would give $T\sim 0.024$, which is still higher than the measured value of $0.01$. For comparison, a density of $\sim 2{n}_{e}^{{\rm{fi}}}$ would decrease the transmission to $0.5\,\%$.

Since the plasma density cannot exceed ${n}_{e}^{{\rm{fi}}}$, then another process must be responsible for the reduction of probe light transmission in the experiment. The simple plasma slab model shows that for ${n}_{e}\gg {n}_{c}$, increasing the plasma thickness $d$ while keeping the product ${n}_{e}d$ constant decreases the transmission. Therefore, our measurements confirm that significant plasma expansion already occurs on sub-ps timescales, and thus needs to be considered in the modeling.

## Discussion

Comprehensive numerical simulations were performed to explain the experimental findings. We investigated different interaction models to compute the time-dependent free electron density ${n}_{e}(t)$. As the transmission profiles in the experiment were found to be insensitive to the peak intensity variation and the plasma forms during the steep rising edge of the laser pulse, the laser intensity profile is described by $I={I}_{\rm{peak}}\,\exp (-\left|{t}_{{\rm{pump}}}\right|/277{\,\rm{fs}})$ in the simulations with *I*_peak_ = 10^15^ W/cm^2^. The transmission of a probe plane wave propagating through the generated plasma with density ${n}_{e}(z,t)$ was calculated to compare the simulation results to the experimental measurements. Further details of our computational methodology are provided in the section Materials and methods.

In a first attempt, the interaction was simulated using the one-dimensional (1D) PIC code SMILEI^28^, considering a cold DLC foil interacting with the pump pulse (simulation parameters are given in Materials and methods). Figure 2e (dashed lines) shows the resulting transmission profiles using the electron densities obtained from the SMILEI PIC code alone. For all target thicknesses, the transmission drops quickly with $\tau \le 100{\,\rm{fs}}$, i.e., much faster than experimentally measured. In addition, Fig. 2f shows that plasma formation (ionization) starts at ${t}_{{\rm{pump}}}\sim -1{\,\rm{ps}}$ where $I\sim \!{10}^{13}\,{\rm{W}}/{{\rm{cm}}}^{2}$, which is the intensity threshold for photo-ionization of carbon atoms. This ultrafast transition from a cold target to a highly overdense plasma, i.e., $> 100{n}_{c}$ over a time interval $< 100{\,\rm{fs}}$, corresponds to the abrupt drop in the predicted probe transmission.

This discrepancy with the experiment can be attributed to the inadequate description of the pristine target. A PIC code treats the target as an ensemble of individual carbon atoms, which does not account for the solid state. Hence, only the ionization of the individual atoms is considered. However, DLC may already be ionized as a solid carbon foil at lower energy. Indeed, the target is a semiconductor with a band gap of $\sim \!1.1{\,\rm{eV}}$. This energy value is much lower than the first ionization energy of carbon atoms $\sim \!11.3{\,\rm{eV}}$. The plasma is, thus, expected to form earlier and at lower laser intensities already. Therefore, the actual ionization dynamics of the solid foil needs to be included in the modeling.

To correctly account for ionization in solids, a simulation was carried out with a solid-state interaction (SSI) model adapted from Ref. ^45^. The ionization is described by solving multiple rate equations^46^ (details are given in Materials and methods). This model was already used to successfully interpret an experiment of laser-induced plasma formation from a dielectric solid presented in Ref. ^42^. The SSI simulations take into account the complete pump laser’s temporal intensity profile (Fig. 1a, solid red line), yielding the temporal evolution of the plasma density ${n}_{e}(t)$ as shown by the violet solid triangles in Fig. 2f. As expected, significant plasma generation occurs in the steep rising edge ${t}_{{\rm{pump}}} > -\!\!3{\,\rm{ps}}$. By assuming homogeneous, non-expanding plasmas with thicknesses corresponding to the target foils, the transmission profiles can be computed and are shown in Fig. 2e (solid triangles). The SSI model stops at ${t}_{{\rm{pump}}}\sim -2.3{\,\rm{ps}}$ at the rising edge where the maximum density ${n}_{e}\sim 20{n}_{c}$ is reached. This model was developed for dielectric materials such as fused silica $({\rm{Si}}{{\rm{O}}}_{2})$ with a high band gap $\sim \!9{\,\rm{eV}}$ and a maximum density ${n}_{e} \sim 20{n}_{c}$.

The results in Fig. 2e (solid triangles) show that the transmission dynamics is not fully described by this model alone, as the total opaqueness $T\sim 0$ is not reached at ${t}_{{\rm{pump}}}\sim -2.3{\,\rm{ps}}$ for none of the target thicknesses investigated. Nevertheless, we observe a significantly slower transmission decrease in the SSI model compared to the previous PIC results in Fig. 2e (dashed lines), closer to our experimental observations. Furthermore, the electron density evolution in Fig. 2f indicates that ionization starts earlier and, thus, at lower intensities than predicted by the PIC code alone, involving atomic ionization rates.

### Solid-state and kinetic plasma description: Two-step model

To overcome the limitations of both models and provide a better description of the target dynamics, we propose a combination of the SSI model at earlier times and the PIC description at later times. On the one hand, the SSI model describes the laser interaction with solids. On the other hand, the PIC code correctly handles the kinetics of a highly overdense plasma. We will refer to the combination of SSI and PIC as the two-step interaction (TSI) model. In the TSI model, the simulation starts with the SSI model and is continued by a PIC simulation after an overdense plasma is formed. As the PIC description considers only free particles such as electrons, ions, and atoms, a reasonable switching point is when the melting state of DLC is reached (Further discussion regarding the switching point is provided in Supplementary materials). At the melting point, the band structure disappears, and the ions start to be free.

**Fig. 3:**
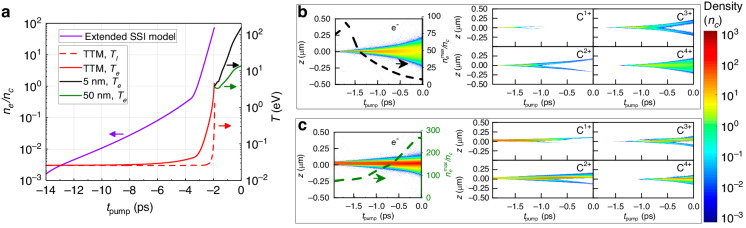
Computed plasma properties during the interaction from the TSI model for DLC foils of 5 and 50 nm thicknesses. Here, the pump laser propagates in positive z-direction. **a** Time-dependent electron density from the SSI model, lattice (${T}_{l}$), and electron (${T}_{e}$) temperatures from the TTM model for all thicknesses and ${T}_{e}$ for 5 and 50 nm thicknesses from PIC simulations. **b** and **c** show the spatiotemporal dynamics of the electron and the carbon ion densities for 5 and 50 nm thick foils, respectively. The black and green dashed lines correspond to maximum electron densities ${n}_{e}^{\max }$ as a function of ${t}_{\rm{pump}}$ for 5 nm and 50 nm thicknesses, respectively

A semiconductor under femtosecond laser irradiation may undergo non-thermal melting^47,48^. In contrast to thermal melting, a significant fraction of the electrons is promoted abruptly from the valence band to the conduction band. Consequently, the lattice bonds are rapidly weakened, and the ions or atoms start to move before reaching the thermal melting point. This scenario may occur as an exponentially increasing laser intensity continuously irradiates the DLC. The non-thermal melting may start at ${n}_{e}\sim 10{n}_{c}$^47^, the threshold for Si, a semiconductor with a band gap of $\sim \!1.12{\,\rm{eV}}$ similar to DLC. Additionally, ions require a few $100{\,\rm{fs}}$ to be entirely free^47^. Therefore, to ensure an initial plasma state composed of entirely free ions, as assumed in the PIC description, we extrapolate the SSI model to ${n}_{e}^{m}\sim 70{n}_{c}$ so that the PIC simulation starts about $0.5{\,\rm{ps}}$ after the beginning of the melting process. In the SSI model, because DLC has a much smaller band gap $(\sim \!1.1{\,\rm{eV}})$ than fused silica for which the SSI model was originally developed, further ionization can occur from the lower bands of the solid state band structure. Thus, a higher electron density may be produced. Therefore, we assume validity of the model up to ${n}_{e}\sim 70{n}_{c}$ for our configuration, and highly overdense plasmas may be obtained from our extended SSI model. However, going beyond this value would certainly break the validity of the model, e.g., due to the ionization of the inner shells of carbon atoms, a process that is not adequately described by the extended SSI model. To bridge the SSI ionization dynamics to the PIC simulations when non-thermal melting occurs, electron and lattice temperatures, ${T}_{e}$ and ${T}_{l}$, respectively, must be determined. Following Refs. ^42,45^ and references therein, we use a standard two-temperature model (TTM). The results shown in Fig. 3a indicate that, at the melting, ${n}_{e}^{m}$ is reached at ${t}_{{\rm{pump}}} \sim -1.93{\,\rm{ps}}$, corresponding to an intensity of $I \sim {10}^{12}\,{\rm{W}}/{{\rm{cm}}}^{2}$ and ${T}_{e}^{m} \sim 4.6{\,\rm{eV}}$ and ${T}_{i}^{m} \sim 0.34{\,\rm{eV}}$. Further details on the TSI parameters are provided in Materials and methods.

Calculated transmission dynamics using the densities ${n}_{e}\left(z,t\right)$ from the TSI model are shown as solid lines in Fig. 2e for the different target thicknesses. All transmission profiles reach $T\sim 0$ in the PIC stage of the model, where plasma expansion is considered. The characteristic times $\tau$ of the transmission dynamics are a few hundreds of fs, which is in good agreement with the measurements as shown in Fig. 2a-d. It is worth noting that extrapolating the SSI model to ${n}_{e}\sim 70{n}_{c}$ shows an excellent agreement with the experiment, further validating the application of the SSI model to materials with a lower band gap such as DLC. The extended SSI domain is indicated by the shaded gray region in Fig. 2e and Fig. 2f. Some slight discrepancies between the TSI results and our measurements can be observed, such as a higher transmission predicted by the TSI model for the PIC results with the thinnest foils and the reverse behavior for the 50 nm case. Possible reasons may be the experimental target thickness uncertainty of about 20 % or a systematic underestimation of the ionization yield and thus *n*_e_ in the PIC simulations due to the abrupt switch to a pure particle description. Besides, an overestimation of *n*_e_ in the SSI description can also be attributed to an inhomogeneous ionization likely occurring for thicker foils because *l*_s_ is shorter than the plasma thickness. This inhomogeneity is currently not taken into account in the first part of the TSI model. Moreover, during its transition from solid to plasma, the target passes through the highly non-linear and – for our conditions – ultrafast regime of warm dense matter (WDM), which neither SSI nor PIC models adequately describe. Considering and mitigating these limitations is beyond the scope of this work. Nevertheless, our TSI approach confirms that an ultrafast solid-to-overdense plasma transition occurs in the experiments. To correctly describe this early stage of the interaction, both the solid properties (SSI model) and the plasma properties (PIC approach) are essential, and only the combination of both models yields an accurate description of the experimental results.

### Comparison of modeling methods: SSI, PIC, and TSI

In Table 1 we summarize the transmission results obtained, characterized by the transmission time τ, from the different modeling methods and their comparison with the experimental results for all target thicknesses.

**Table 1:** Transmission time τ obtained from the experiments and all models

DLC foil (nm)	*τ*_exp_ (ps)	*τ*_PIC_ (ps)	*τ*_SSI_ (ps)	*τ*_TSI_ (ps)
5	0.47 ± 0.13	0.1	∞	1.02
10	0.63 ± 0.08	0.05	∞	0.82
20	0.37 ± 0.14	0.03	∞	0.55
50	0.52 ± 0.23	0.03	∞	0.44

τ is the time required for the transmission to drop from 90 % to 10 % relative transmission

As highlighted previously, the transmissions obtained from PIC simulations starting from a cold DLC foil drop much faster than in experiment with ${\tau }_{{\rm{PIC}}}$ $\le 100{\,\rm{fs}}$. No meaningful transmission time ${\tau }_{{\rm{SSI}}}$ could be extracted, because the full transmission dynamics could not be described using the SSI model alone (cf. Figure 2e, solid triangles). However, when combining the extended SSI model and PIC in the TSI model, ${\tau }_{{\rm{TSI}}}$ shows good agreement with the experimental values. Although none of the two models (SSI or PIC) alone could explain the experimental observations, they are, however, complementary, as each of them includes necessary fundamental processes to describe an ultrafast laser-solid interaction. On the one hand, the initial stage of the interaction, i.e., ionization of a solid, can only be accurately described using the SSI model. Such a description is not possible in the PIC description as the latter only considers the laser interaction with free particles. On the other hand, a low transmission at later times indicates, as discussed previously, a highly overdense and/or expanding plasma. Such plasma cannot be described by the SSI model, but the PIC description accounts for plasma kinetics (expansion) and the inner shell ionization process. Therefore, the ultrafast solid-to-overdense plasma transition as observed in our experiments can only be accurately described using the TSI model.

### Initial plasma expansion and interplay of ionization processes

To emphasize the role of the plasma expansion, Fig. 3b, c shows the computed spatiotemporal evolution of the plasma properties for the 5 nm and the 50 nm-thick foils. For the thinnest foil in Fig. 3b, ${n}_{e}\left(z,t\right)$ exhibits a strongly expanded profile at the end of the simulation, caused by the rapid heating of the plasma as the intensity approaches the peak. At ${t}_{{\rm{pump}}}=0{\,\rm{ps}}$, the plasma thickness with density ${n}_{e}\ge {n}_{c}$ is estimated to be ∼ 300 nm, about 60 times the original target thickness. Besides, the relatively low maximum density of ${n}_{e}\sim 7{n}_{c}$ highlights the importance of the plasma expansion for lowering the probe transmission. Furthermore, the density profile evolves symmetrically in time, in contrast to the strong asymmetry observed for the 50 nm-thick foil shown in Fig. 3c, where a high-density region reaching a maximum of $\sim 270{n}_{c}$ with extended low-density drops at the target front and back side, while the drop is steeper at the back. These results point out that, on the one hand, our measurements evidenced the plasma expansion for the thinnest foil, and, on the other hand, applying the TSI model is crucial for correctly describing the interaction in its initial phase. Such detailed knowledge of the target evolution is paramount for matching the laser contrast conditions to the target (or plasma) thickness to achieve efficient laser-driven ion acceleration.

Finally, the interplay of fundamental processes such as ionization and collisions, which eventually determine the target properties at the peak arrival, is accessible by using our experimental measurements and comparison to our modeling strategy. During the laser-induced solid-to-plasma transition investigated in this work, the free electrons are produced in the SSI step by the multi-photon ionization (MPI) process in the solid state. Due to the low electron temperature during this step (${T}_{e}\, <\, 5{\,\rm{eV}}$) inferred from the TTM, cf. Figure 3a, collisional ionization (CI) is negligible, estimated to be $\sim \!1 \,\%$. In fact, although ${n}_{e}\sim 70{n}_{c}$ at ${t}_{{\rm{pump}}}\sim -2$ ${\rm{ps}}$, the laser intensity $I\sim {10}^{12}\,{\rm{W}}/{{\rm{cm}}}^{2}$ is not sufficient to heat the electrons to induce a significant number of ionizing collisions. In the PIC step, however, collisions occur as free electrons are available and heated by the exponentially increasing laser intensity. Consequently, CI starts at a lower intensity than the threshold intensity $I\sim {10}^{13}\,{\rm{W}}/{{\rm{cm}}}^{2}$ for photoionization of carbon ions and becomes quickly dominant. The abrupt behavior of CI, being negligible at the end of SSI and being dominant at the beginning of the PIC phase, suggests that CI starts to become dominant during the highly non-linear and ultrafast WDM transition, which our TSI model does not describe. Therefore, despite being in an intensity range suitable for photo-ionizing carbon ions, this ionization process does not play a role in our experiments. Simulations without photoionization in SMILEI do not alter the TSI results shown in Fig. 2, and thus confirm this interpretation. While the ionization charge state of the pre-plasma is usually based on assumptions when modeling relativistic laser-matter interactions, in this work, gaining insight into the ionization processes naturally leads to the detailed knowledge of different ion species and their dynamics in the plasma. For example, the final average charge state$\,{C}^{4+}$ shown in Fig. 3b, c could not have been predicted without considering collisions and CI being the dominant ionization mechanism.

### Conclusions

In summary, our investigation sheds light on the sub-picosecond transition from a solid target to a highly overdense plasma (${n}_{e} > 100{n}_{c}$) produced with nm-thin DLC foils during the laser rising edge with intensities increasing up to $I\sim {10}^{16}\,{\rm{W}}/{{\rm{cm}}}^{2}$. Even though this stage of pre-plasma formation is crucial for the conditions for the subsequent ion acceleration during a relativistic laser-thin foil interaction, this transition has neither been studied in detail in simulations nor detected in experiments. We demonstrated an all-optical single-shot technique that characterizes the complete evolution of the target. Because our technique relies on optical tunneling, accessing the overdense plasma regime is possible. Our findings indicate that a correct description of the target transition from a solid to a plasma state is crucial for understanding the plasma evolution in laser-solid interactions, which is of paramount importance not only for pre-plasma characterization in the high-power laser regime but also for laser ablation in the low-power laser regime.

Our single-shot NIR probe transmission measurements evidence a non-negligible plasma expansion that can significantly reduce the probe intensity tunneling through very thin foils. We develop a general picture of the evolution of the plasma by employing a two-step interaction model comprising a combination of a solid-state interaction model and a PIC code. A detailed description of the pre-plasma properties before the peak arrival is achieved, going well beyond the previous modeling of high-intensity laser-matter interactions.

Our approach can readily provide a detailed description of the plasma formed by pre-expanding a thin foil using a controlled pre-pulse, usually in the intensity range studied in this work. Besides being of fundamental interest, such insight is crucial to finding the matching laser-target conditions required for the RIT-based acceleration regime. Our experimental findings and the application of our modeling approaches might therefore contribute to making laser-accelerated ion technologies usable for societal applications. Finally, our method can be extended to thicker foils that exhibit high initial transmission, such as dielectrics. In this case, the TSI model must be modified accordingly to consider the target inhomogeneity during the interaction. A relatively simple approach could be to solve the one-dimensional Helmholtz equation coupled to the TTM model in the SSI step. Such a modified TSI model could be also used to describe the ionization and plasma dynamics of thick nontransparent targets, where the initial plasma is produced on the front surface only.

## Materials and methods

### Laser system and pulse characterization

The experiments were carried out using the all-diode pumped high-power laser system POLARIS^49^, operated by the Helmholtz-Institute Jena and the Institute of Optics and Quantum Electronics in Jena. The temporal intensity contrast of the pump laser, as measured with a third-order cross-correlator (Amplitude, Sequoia), is shown in Fig. 1a. The profile is well described by the two fitting curves in red. The indicated artifact at ${t}_{{\rm{pump}}} \sim -5{\,\rm{ps}}$ is due to a post-pulse induced by a glass wafer inserted into the beam path in the interaction chamber for debris shielding, hence not affecting the interaction, and is therefore ignored throughout our analysis.

Particularly relevant is the steep rising edge in the time window $-3.7{\,\rm{ps}}\le {t}_{{\rm{pump}}}\le -0.2{\,\rm{ps}}$, described by the contrast ratio ${\rm{CR}}=\exp (-\left|{t}_{{\rm{pump}}}\right|/277{\,\rm{fs}})$ with an intensity profile $I={I}_{{\rm{peak}}}\times {\rm{CR}}$, where ${I}_{{\rm{peak}}}$ is the intensity of the peak characterized by $\sim 150{\,\rm{fs}}$ Full Width at Half Maximum (FWHM) pulse duration. In the experiments, the peak intensity is reduced to ${I}_{{\rm{peak}}} \sim {10}^{15}\,{\rm{W}}/{{\rm{cm}}}^{2}$ by inserting a half-inch aperture into the beam path of the few J-energy, 140 mm-diameter, and linearly polarized pulses centered at ${\lambda }_{p}=1030{\,\rm{nm}}$ before being focused with an off-axis parabola (300 mm-focal length) at normal incidence on the DLC foil. Thus, the relative contrast profile is kept similar to that for the relativistic high-intensity pulses. The resulting $\approx 3{\,\rm{mJ}}$ energy pump pulses are focused to a spot showing an Airy pattern (see the inset in Fig. 1b) with a $\approx \,\!40\,{\upmu} \rm{m}$ FWHM diameter containing $\sim \,\!60 \,\%$ of the pulse energy after the aperture. The experiments were carried out inside a vacuum chamber at a pressure of about 10^−5^ mbar.

### Target

The diamond-like carbon (DLC) targets used in this experiment are free-standing foils of pure carbon (Micromatter Technologies^TM^ Inc., with a mass density of ${\rho }_{{\rm{DLC}}}=2.15\,{\rm{g}}/{{\rm{cm}}}^{3}$). The foils were initially coated on a 1 mm-thick borosilicate glass substrate (BK7) using the pulsed laser deposition technique^50^. A thin layer of a release agent is added between the foil and the substrate, which facilitates the transfer of the pure DLC foils to the target holder. The DLC foil is an amorphous semiconductor^51^, characterized by an electronic band structure with a band gap of $\sim 1.1\,{\rm{eV}}$. The latter was estimated using the Tauc method^52^. The target refractive index is given by ${\eta }_{{\rm{DLC}}}={n}_{{\rm{DLC}}}+i{\kappa }_{{\rm{DLC}}}$ where ${n}_{{\rm{DLC}}}\approx 2.65$^53^ and the extinction coefficient ${\kappa }_{{\rm{DLC}}}\approx 0.5$, which is obtained from a wavelength-dependent transmission measurement carried out using a Shimadzu Solid Spec-3700-spectrometer. The carbon ionization energies are 11.3, 24.4, 47.8, 64.5, 392, and 490 eV.

### Single-shot space and time-resolved probe transmission diagnostic

The plasma dynamics are investigated by longitudinally irradiating a $\sim 100\,{\upmu}\rm{m}$-extended region of the interaction with $p$-polarized broadband ($\Delta \lambda \approx 150{\,\rm{nm}}$ centered at $\lambda \approx 840{\,\rm{nm}}$ under an incidence angle of $\alpha =37^{\circ}$) and $\approx 12{\rm{\mu J}}$-energy probe pulses produced in a Non-collinear Optical Parametric Amplifier (NOPA)^54^. When optimally compressed, the probe pulses have a duration of $\approx \!14{\,\rm{fs}}$. However, by applying a positive chirp to the pulses, their duration is stretched to $\sim 6{\,\rm{ps}}$ so that their different wavelength components arrive at different interaction times. With this approach, the plasma formation and evolution can be recorded within a single shot, achieving a sub-ps time resolution^44^. An extent of $\Delta x\approx 3\,{\upmu} \rm{m}$ of the interaction region is imaged onto the entrance slit of the 1D-spatially resolving spectrometer. The relative timing between POLARIS-main and NOPA-probe pulses (seeded by the same oscillator) can be adjusted using a delay stage with sub-ps resolution. We measure the relative probe transmission through the plasma ${T}_{r}=T/{T}_{0}$, where ${T}_{0}$ and $T$ are the measured transmission values without and with the interaction induced by the pump pulse, respectively. The conversion of the probe wavelength to time is calibrated using an additional pre-pulse with an adjustable time delay. The measured absolute transmission $T$ in Fig. 2a–d are obtained as the lineout at $y=0\,{\upmu {\rm{m}}}$ averaged by $3\,{\upmu {\rm{m}}}$. The measurements in the same figure are averaged over four shots with intensities in the range $I \sim {10}^{15}-{10}^{16}\,{\rm{W}}/{{\rm{cm}}}^{2}$ for each foil thickness.

### Modeling

#### Analytical model for optical tunneling

We assume a $p$-polarized probe plane wave at an angle of incidence of $\alpha =37^{\circ}$ at the overdense and homogeneous plasma slab of thickness $d$ with the dielectric function $\varepsilon\, <\, 0$. Then, exploiting Maxwell’s boundary conditions^55^ at the front and rear sides of the slab yields the transmission

1 $${T}=\frac{4{e}^{-4\pi \gamma d/\lambda }}{\left(1+{C}^{2}\right)\left(1+{e}^{-8\pi \gamma d/\lambda }\right)+2\left(1-{C}^{2}\right){e}^{-4\pi \gamma d/\lambda }}$$

with $\gamma =\sqrt{{\sin }^{2}\alpha -\varepsilon }$, $C=\frac{{\gamma }^{2}-{\varepsilon }^{2}{\cos }^{2}\alpha }{2\varepsilon \gamma \cos \alpha }$ and $\lambda$ being the central probe wavelength. For a highly overdense plasma, we can assume $\varepsilon \approx 1-{n}_{e}/{n}_{c}$.

#### Numerical simulations

We used two complementary interaction models, discussed in detail below, to compute the time-dependent free electron density ${n}_{e}(t)$. As the transmission profiles in the experiment were found to be insensitive to the peak intensity variation and the plasma forms during the steep rising edge of the laser pulse, the laser intensity is described by $I={I}_{\rm{peak}}\,\exp (-|{t}_{{\rm{pump}}}|/277\,{\rm{fs}})$ in the simulations, and ${I}_{\rm{peak}}={10}^{15}\,{\rm{W}}/{{\rm{cm}}}^{2}$. The transmission of a probe plane wave propagating through the generated plasma with density ${n}_{e}(z,{t})$ was calculated by solving Maxwell’s equations. To this end, the matrix method presented, e.g., in Refs. ^56,57^ was adapted. The Drude model is used to compute the complex dielectric function $\varepsilon$ expressed as a function of ${n}_{e}$ as

2 $${\rm{\varepsilon }}={\eta }_{{\rm{DLC}}}^{2}-\frac{{n}_{e}}{{n}_{c}}\left(1+i\frac{{\nu }_{c}}{\omega }\right)$$

with ${\eta }_{{\rm{DLC}}}$ being the refractive index of the pristine target (see target section.); the temperature-averaged electron-ion collision frequency was chosen as ${\nu }_{c}=5\times {10}^{14}\,{{\rm{s}}}^{-1}$, consistent with what is discussed and used in the two-temperature model section; $\omega$ is the angular frequency of the probe.

##### Particle-in-cell simulation (PIC)

We use the one-dimensional (1D) PIC code SMILEI^28^. The relevant implemented processes include multiphoton ionization (MPI), field ionization (tunneling ionization, TI), and collisional ionization (CI) for atoms, and binary collisions between electrons and ions. In the simulation box of $L=1{\,{\upmu {\rm{m}}}}$ length with a cell size of $\Delta z=0.156{\,\rm{nm}}$, the target was modeled as a slab of cold carbon atoms at solid density ${n}_{a}=62{n}_{c}$, positioned at $z=0\,{\upmu {\rm{m}}}$ (cf. Fig. 3b, c). The pump laser with a central wavelength ${\lambda }_{p}=1030{\,\rm{nm}}$ enters the box from $z \,< \,0$. Its temporal intensity envelope follows the steep rising edge mentioned above. The temporal resolution was $\Delta t=5\times {10}^{-4}\,{\rm{fs}}$. 2000 macro-particles per cell were initialized in all the simulations.

##### Solid-state interaction model (SSI)

To correctly account for ionization in solids, we use a solid-state interaction (SSI) model adapted from Ref. ^45^. In this model, the ionization is described by solving state-of-the-art multiple rate equations^46^ where the target band structure is described by a set of states accounting for the electron dynamics in the conduction band. An electron density ${{n}}_{i}$ is associated with each state (where $i\in 0,1,2$), and the coupled system reads

3 $$\frac{\partial {n}_{0}}{\partial t}={W}_{{\rm{PI}}}+2\widetilde{\alpha }{n}_{2}-{W}_{1}{n}_{0}-{n}_{0}/{\tau }_{r}$$

4 $$\frac{\partial {n}_{1}}{\partial t}={W}_{1}{n}_{0}-{W}_{1}{n}_{1}-{n}_{1}/{\tau }_{r}$$

5 $$\frac{\partial {n}_{2}}{\partial t}={W}_{1}{n}_{1}-\widetilde{\alpha }{n}_{2}-{n}_{2}/{\tau }_{r}$$

The first conduction state, ${n}_{0}$ is filled with a ${W}_{{\rm{PI}}}$ rate (MPI or TI depending on the intensity) obtained from the Keldysh theory^58^. This stage describes the primary photo-ionization process. Each conduction state is bridged by one-photon absorption similar to the mechanism of inverse Bremsstrahlung absorption through the rate ${{W}}_{1}=3.5\times {10}^{-7}\,{E}_{L}^{2}$ in units of ${{\rm{s}}}^{-1}$, where ${E}_{L}$ is the laser electric field in units of ${\rm{V}}\,{{\rm{m}^{-1}}}$. The SSI model includes CI and possible electron avalanche ionization, as highly energetic electrons in the conduction band may transfer a fraction of their energy by collisions with electrons in the valence band. The last state corresponds to the minimum energy required to induce impact ionization (i.e., at least $1.5$ times the bandgap^46^) with the rate $\widetilde{\alpha }={10}^{15}\,{{\rm{s}}}^{-1}$. Conduction electrons can also recombine within a characteristic timescale of ${\tau}_{r}=1\,{\rm{ps}}$. These parameter values have already been used in various studies compatible with the present conditions^59–62^. The free electron density is ${n}_{e}={n}_{0}+{n}_{1}+{n}_{2}$. Because the emptying of the valence band is not accounted for, ${n}_{e}$ can exceed tens of critical plasma densities. This approach remains valid as long as the band structure remains intact, that is, before melting occurs.

##### Two-temperature model (TTM)

Following Refs. ^42,45^ and references therein, we use a standard two-temperature model (TTM),

6 $${C}_{e}\frac{\partial {T}_{e}}{\partial t}=\frac{\partial U}{\partial t}-\frac{3}{2}{k}_{B}\frac{\partial {n}_{e}}{\partial t}{T}_{e}-G\left({T}_{e}-{T}_{l}\right)$$

7 $${C}_{l}\frac{\partial {T}_{l}}{\partial t}=G\left({T}_{e}-{T}_{l}\right)$$

The heat capacities are ${C}_{e}=3{n}_{e}{k}_{B}/2$ and ${C}_{l}=3{n}_{a}{k}_{B}/2$. The electron-ion energy exchange factor $G$ is evaluated by $G={C}_{e}{\nu }_{c}{m}_{e}/{m}_{a}$. ${n}_{a}$ and ${m}_{a}$ are the carbon atomic density and mass, respectively. The electron-to-ion mass ratio weights the collision frequency to account for energy exchange (${\nu }_{c}$ accounts for momentum transfer). The source term is evaluated with the Drude model:

8 $$\frac{\partial U}{\partial t}=\frac{{e}^{2}{n}_{e}{\nu }_{c}}{{m}_{e}({\omega }^{2}+{\nu }_{c}^{2})}{E}_{L}^{2}$$

where ${\nu }_{c}={\nu }_{{\rm{ph}}}$ is the electron-phonon collision frequency since the collisions are driven mainly by phonons in the solid state. It then reads ${\nu }_{{\rm{ph}}}={\nu }_{{\rm{ph}}0}{T}_{l}/{T}_{0}$, and ${\nu }_{{\rm{ph}}0}$ is the electron-phonon collision frequency at room temperature ${T}_{0}=300{\,\rm{K}}$. It is set to ${\nu }_{{\rm{ph}}0}={10}^{14}\,{{\rm{s}}}^{-1}$ ^60^. ${\nu }_{c}$ is limited to $5\times {10}^{15}\,{{\rm{s}}}^{-1}$ to account for the upper value of the collision frequency imposed by the electron mean free path^45^.

##### Two-step interaction model (TSI)

In our TSI model, the time-dependent electron density is first computed using the SSI model above, followed by the PIC simulation which starts with a homogeneous plasma slab with a density ${n}_{e}^{m}\approx 70{n}_{c}$ of the initial target thickness. The electrons and carbon ions species are initialized with Maxwell-Boltzmann distribution functions with temperatures ${T}_{e}^{m}$ and ${T}_{i}^{m}$, respectively, computed in the TTM. Since ${n}_{e}^{m}$ exceeds the carbon solid atomic density ${n}_{a}=62{n}_{c}$, the plasma is modeled partially ionized with a mixture of single and double ionization states of carbons ${C}^{1+}$ and ${C}^{2+}$ with ${n}_{{C}^{1+}}=54{n}_{c}$ and ${n}_{{C}^{2+}}=8{n}_{c}$, respectively. In the low-intensity range with $I\, <\, {10}^{12}\,{\rm{W}}/{{\rm{cm}}}^{2}$ where the SSI description holds, we expect full single ionization of carbon atoms by MPI reaching the density ${n}_{{C}^{1+}}={n}_{a}$ before a significant number of ${C}^{2+}$ is produced. Collisions are of minor importance because electrons are only moderately heated by the laser in this intensity range. With increasing intensity, a fraction of these ions is further ionized to ${C}^{2+}$ to reach ${n}_{e}^{m}$. The other simulation parameters are kept the same as in the section PIC above.

### Supplementary information

Supplementary Information for Optical Probing of Ultrafast Laser-Induced Solid-to-Overdense-Plasma Transitions

## Data Availability

The experimental and simulation data that support the findings of this work are available from the corresponding author upon reasonable request.
