# Supplementary material for: Optical probing of ultrafast laser-induced solid-to-overdense-plasma transitions
Source: Light Sci Appl. 2024 May 8;13:109. doi: 10.1038/s41377-024-01444-y (PMC11079011; doi:10.1038/s41377-024-01444-y)

Supplementary Information for **Optical Probing of Ultrafast Laser-Induced Solid-to-Overdense-Plasma Transitions**

Yasmina Azamoum^1, 2, ∗^, Georg Alexander Becker^2^, Sebastian Keppler^1, 2^, Guillaume Duchateau^3^, Stefan Skupin^4^, Mickael Grech^5^, Fabrice Catoire^6^, Sebastian Hell^2^, Issa Tamer^1,2^, Marco Hornung^1, 2^, Marco Hellwing^2^, Alexander Kessler^1^, Franck Schorcht^1^, and Malte Christoph Kaluza^1, 2^

^1^Helmholtz Institute Jena, Fröbelstieg 3, 07743 Jena, Germany.
^2^Institute of Optics and Quantum Electronics, Friedrich-Schiller-Universität Jena, Max-Wien-Platz 1, 07743 Jena Germany.
^3^CEA-CESTA, 15 Avenue des Sablières, CS60001, 33116 Le Barp Cedex, France.
^4^Institut Lumière Matière, UMR 5306 - CNRS, Université de Lyon 1, 69622 Villeurbanne, France.
^5^LULI, CNRS, CEA, Sorbonne Université, Institut Polytechnique de Paris, Palaiseau, France.
^6^Université de Bordeaux-CNRS-CEA, CELIA, UMR 5107, Talence, France.

*Corresponding author: [yasmina.azamoum@uni-jena.de](mailto:yasmina.azamoum@uni-jena.de)

**The transmittance of different thickness DLC foils**

The absolute values of the pristine target transmission considered in Fig. 2 of the primary manuscript (high T plateau region, blue line) were deduced from a separate measurement using a commercial Shimadzu Solid 3700-Spectrometer. These measurements were made with the DLC foils coated on a 1 mm thick borosilicate (BK7) glass substrate at normal incidence. This geometry was imposed by the fixed configuration of the spectrometer. The results, in the probe wavelength range (700 – 900 nm) window, are shown in Figure 1sup.

Fig. S1 shows that the target dispersion is very low as the transmission variation is < 8 % in the probe wavelength range. In addition, in our calculations, the plasma dispersion is also found to be negligible in this wavelength range; see Fig. S2. Therefore, it is sufficient to discuss the transmission values at the chosen probe center wavelength of 800 nm.

**Fig. S1:** Measured transmission at normal incidence through the pristine DLC foils coated on a 1 mm thick borosilicate (BK7) glass substrate, using a commercial Shimadzu Solid 3700-Spectrometer (solid lines, for different DLC thicknesses 5 nm: black, 10 nm: red, 20 nm: blue and 50 nm: green). Keeping the same color code for the DLC foil thickness, the open and solid squares are the computed DLC foil with the substrate and pure DLC foils at 800 nm, respectively.


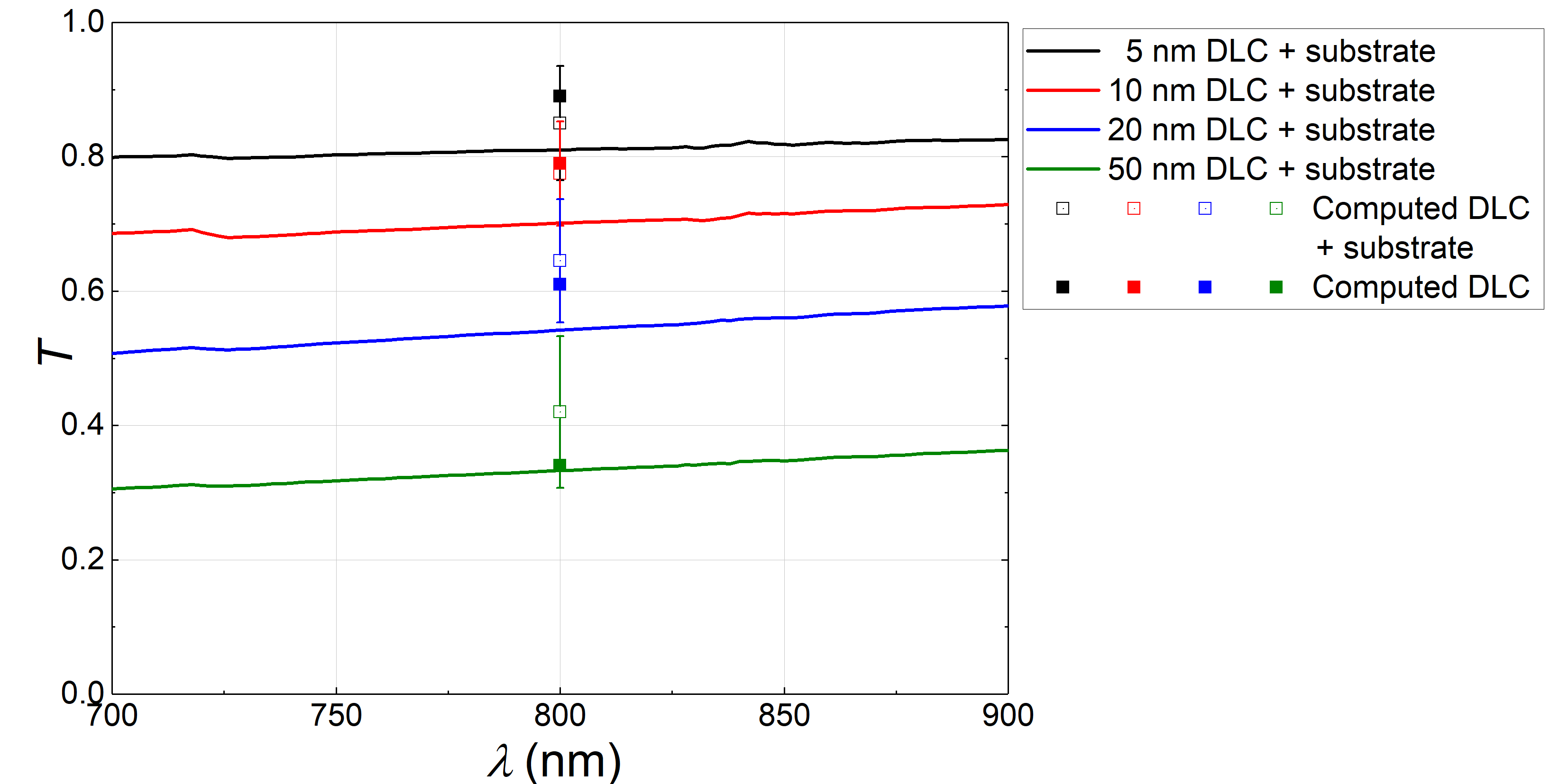


To estimate the transmission values of the pristine free-standing DLC foils used in the experiments, we calculated the transmission of the composed DLC foil and the BK7 substrate by considering the multiple reflections at the interfaces with the dielectric constants of *ε*_DLC_ = 6.7725 + i 2.65 (given in the primary manuscript) and *ε*_BK7_ = 2.2824 + i 2.7997e-8 (from the SCHOTT Zemax catalog, see refractiveindex.info). The results are shown in open squares in Fig. S1. The error bars originate from the high amplitude of the oscillations due to the Fabry-Perot effect in the glass substrate. The measured transmissions for the various DLC foils coated on the substrate lie close or within these theoretical error bars. The systematically lower measured values can be attributed to several factors. In fact, we estimate a systematic error of a few percent due to the baseline of the spectrometer, which results in lower transmission. Furthermore, the uncertainty in the thickness of the DLC thickness (~ 20 %) and the release agent between the foil and the substrate were not taken into account in the calculations.


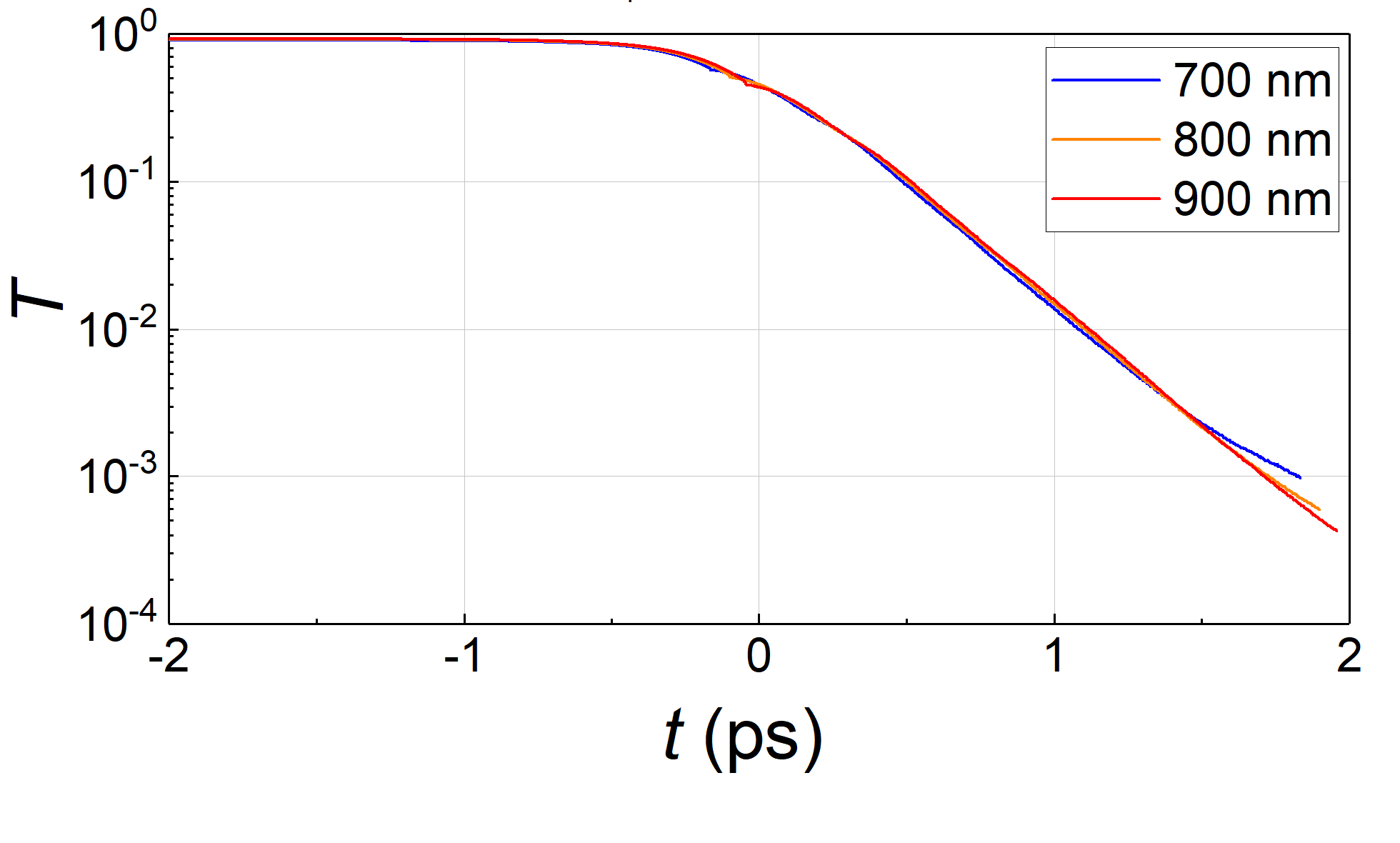


**Fig. S2:** Computed transmission of a plane wave centered at different wavelengths in the probe window (700 nm: blue, 800 nm: orange and 900 nm: red, respectively), using the two-step model for 5 nm-thick DLC foil. The different plots are overlapped at their inflection point corresponding to relative transmission of 50 %. The time at this point is set to t = 0 ps.

Therefore, we can assume that the pure free-standing DLC foils transmissions at 800 nm in the experiments (before plasma formation) have values close to the computed values depicted in solid squares in Fig. S1. Furthermore, since the p-polarized probe illuminates the target at an angle of incidence of 37 °, the transmission values of the DLC foils in the pristine region in Fig. 2 in the primary manuscript exhibit higher values by a few percent compared to the results of Fig. S1, which were performed at normal incidence.

**Discussion of the connection point in the TSI model**

The two-step interaction model described in section *Solid-state and kinetic plasma description: Two-step model* of the primary manuscript comprises two interaction models (extended SSI and PIC). On the one hand, the initial interaction of the target with the pump laser pulse in its solid state is described by the extended SSI model. On the other hand, the interaction in the highly overdense plasma state is modeled using the PIC code. To switch from SSI to PIC description, one has to choose the connection point based on physical conditions of the target, as discussed in the following.

In Fig. S3, the solid lines describe the TSI model for switching at *n_e_* ~ *70n_c_*, corresponding to the expected fully melted target, as described in detail in the primary manuscript. The dashed and dotted lines correspond to the cases where the fully single ionization state of the target (*n_e_ ~ 62n_c_*), or the validity limit of the original SSI model (*n_e_ ~ 20n_c_*) are reached, respectively. As the connection points for the two latter cases occur earlier in the laser rising edge, a higher transmission is observed in the PIC stage because ionization of the solid state is not accounted for. The best agreement with the measurements is achieved for the latest connection point, taking into account non-thermal melting for DLC being a semiconductor and ensuring fully free ions of the target, in order to fulfill the atomic description in the PIC code.

**Fig. S3**: Computed transmission of the probe from the two-step model during the interaction of the pump pulse with DLC foils of different thicknesses (5 nm: black, 10 nm: red, 20 nm: blue and 50 nm: green). The connection point between the SSI and PIC steps in the TSI model is set to different values: when the target melting point is reached, when full single ionization state of the target is reached, and when the limit of validity of the original SSI model is reached. The corresponding electron densities $n_{e}^{sw}$ are ${70n}_{c}$, ${62n}_{c}$ and ${20n}_{c}$ respectively.


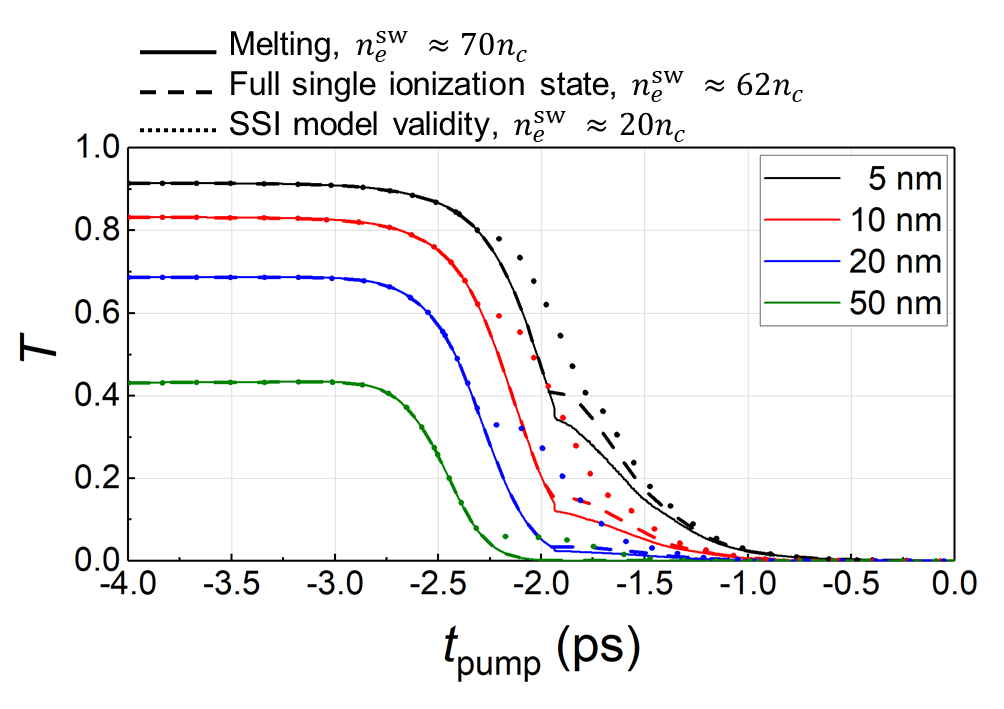

Supplement: Supplementary file 1 — Supplementary Information for Optical Probing of Ultrafast Laser-Induced Solid-to-Overdense-Plasma Transitions [file 41377_2024_1444_MOESM1_ESM.docx]
